# Combined complex electrophysiological interventions due to improved standardization and efficiency: proof of concept

**DOI:** 10.1093/europace/euae014

**Published:** 2024-01-16

**Authors:** Benjamin Berte, Helmut Pürerfellner, Laurent Roten, Sophie Rissotto, Saagar Mahida, Tobias Reichlin, Richard Kobza

**Affiliations:** Heart Center, Hirslanden St Anna, Zentralstrasse 1, 6003 Lucerne, Switzerland; Cardiology Department, Ordensklinikum, Linz, Austria; Cardiology Department, Inselspital Bern, Bern, Switzerland; R and D Department, Johnson and Johnson, Zug, Switerland; Department of Cardiac Electrophysiology, Liverpool Heart and Chest Hospital, Liverpool, UK; Cardiology Department, Inselspital Bern, Bern, Switzerland; Heart Center, Hirslanden St Anna, Zentralstrasse 1, 6003 Lucerne, Switzerland

**Keywords:** Ventricular tachycardia, Atrial arrhythmia, Efficiency, RF ablation

The field of cardiac electrophysiology (EP) is evolving rapidly. Over the years, with the evolution of ablation technologies, including the development of irrigated ablation catheters with contact force sensing, steerable sheaths, 3D mapping systems, multipolar mapping catheters, and pre- and peri-procedural imaging, the treatment of complex arrhythmias with catheter ablation has expanded significantly. Implementation of lean management strategies for complex EP procedures, with standardized, simplified, shorter, and more predictable workflows, has the potential to significantly enhance procedural efficiency.^1–6^

Multiple arrhythmias may co-exist in the same patient. The co-existence of arrhythmias may present unique management problems. For instance, atrial arrhythmias are a common cause of inappropriate ICD therapy.^7^ These inappropriate shocks are potentially responsible for an increased mortality.^8,9^ Eliminating atrial fibrillation (AF) and atrial flutter could reduce appropriate and inappropriate therapy and potentially reduce mortality. Multiple co-existing arrhythmias could be treated during the same procedure, using the same EP material and with standardized workflows. Combinations of ventricular tachycardia (VT) ablation with pulmonary vein isolation (PVI) or cavotricuspid isthmus (CTI) could have potential advantages, including a reduction in the number of procedures, a reduction in complication rates, and more effective utilization of healthcare resources.

The aim of the present study was to analyse the feasibility, safety, and effectiveness of combined complex endocardial only VT ablation procedures with atrial ablation procedures. Ventricular tachycardia ablation was performed using an image integration–guided and substrate-based ablation in sinus rhythm (50 W; QDOT catheter). The MUSIC/inHEART technology was used for image integration as described previously.^2^

Nine patients were included between April 2020 and April 2022 [8 (89%) male; 8 (89%) structural heart disease; age 66 ± 7 years, *Table 1*]. Four (44%) underwent AF ablation and CTI-dependent flutter ablation in addition to VT ablation, 3 (33%) underwent AF ablation in combination with VT ablation, and 2 (22%) had CTI-only ablation in addition to VT ablation. All patients had a history of AF [7 (77%) paroxysmal AF; 2 (22%) persistent AF). One-third of the patients (*n* = 3) had inappropriate defibrillator shocks for rapidly conducted AF. All patients who underwent VT ablation had appropriate therapy (sustained VT with V1− in *n* = 3 and V1+ in *n* = 3), and two patients came with electrical storm. Three patients had inappropriate shocks. Among the cohort of AF ablation patients, three individuals had symptomatic AF, and three patients were ablated prior to CRT upgrade to increase chances of synchronization obtaining biventricular pacing of >98%. Two patients had severe mitral regurgitation, and AF ablation was performed prior to MitraClip. One patient had a tachycardiomyopathy with a LVEF of 35% due to fast persistent AF.

**Table 1:** Data on clinical characteristics, procedural data and safety and follow-up

	Combined EP	MUSIC VT	POWER-PLUS	*P* value
Clinical characteristics				
Total number of patients	9	49	90	
Age, mean (SD) years	66 (7.5)	63 (15)	64.2 (8.9)	0.708
Male, *n* (%)	8 (89)	44 (90)	61 (67.8)	0.009
Paroxysmal AF, *n* (%)	7 (77.8)	NK	64 (71.1)	0.672
Persistent AF (>7 d) *n* (%)	2 (22.2)	NK	26 (28.9)	0.672
BMI, mean (SD) kg/m^2^	26.7 (5)	26.6 (3.7)	26.6 (3.1)	0.997
Hypertension, *n* (%)	3 (33.3)	29 (59)	41 (45.6)	0.188
Structural heart disease, *n* (%)	8 (89)	43 (88)	16 (17.8)	<0.00001
Diabetes, *n* (%)	4 (44.4)	4 (8)	8 (8.9)	<0.00001
CHA_2_DS_2_VAS_C_, median (IQR)	4.0 (4)	4.1 (1.4)	2.0 (3)	<0.00001
LAVI mL/m^2^, mean (SD) mm	46.9 (17.8)	19 (39)	38.9 (5.1)	<0.00001
Inappropriate shocks, *n* (%)	3 (33.3)	NK		–
Appropriate shocks or ATP, *n* (%)	9 (100)	49 (100)		–
Procedural characteristics				
Net procedure time for PVI, median (IQR) min	27 (8)	NA	70 (20)	<0.00001
Procedure time VT, mean (SD) min	140.2 (41.5)	172 (48)	NA	0.062
Steam pop during case	0	1 (4%)	1 (1.1)	–
Char at inspection	0	0	0	–
Ablation time mean (SD)—first to last tag, min	22.8 (4)	80 (37)	32 (12)	<0.00001
RF delivery time LA required, mean (SD) min	6.4 (1.5)	NA	4.5 (1.2)	0.005
RF time VT, mean (SD) min	15.4 (9.1)	31 (17)	NA	<0.001
Overall procedure time, mean (SD) min	163 (22.8)	172 (48)	70 (20)	<0.00001
Safety and effectiveness				
Major complications, *n* (%)	0	1 (2)	0	–
Minor complications, *n* (%)	0	1 (2)	0	–
Recurrence, *n* (%)	2 (22.2)	13 (27)	15 (16.6)	0.380
Mortality, *n* (%)	0	2 (4)	0	–
Appropriate therapy (ATP/shock)	1 (11.1)	NK	NA	–

AF, atrial fibrillation; ATP, antitachycardia pacing; d, days; IQR, interquartile range; LAVI, left atrial volume; min, minutes; mm, millimetres; *n*, number; NA, not applicable; NK, not known; SD, standard deviation; VT, ventricular tachycardia.

The predefined procedure endpoints for AF (pulmonary vein isolation) and VT ablation (CT channel elimination and non-inducibility of any VT) were reached in all nine patients (examples in *Figure 1*). There were no acute procedure-related complications. Skin-to-skin duration was 162.9 ± 40.7 min. The incremental procedure time to complete the atrial ablation was 23 ± 4 min. Of note, atrial fast anatomical mapping is routinely performed as part of the image integration protocol prior to VT ablation. Ablation time (first to last ablation application) for PVI was comparable to the previously reported POWER-PLUS study.^10^ The remaining time of the skin-to-skin duration was counted as the procedural time for VT ablation. There were no significant differences in procedure time for the VT ablation procedure in comparison to the previously reported MUSIC-VT study.^2^ The radiofrequency (RF) time for VT ablation was also not significantly different in our cohort, although we observed a trend towards lower ablation time.

**Figure 1:**
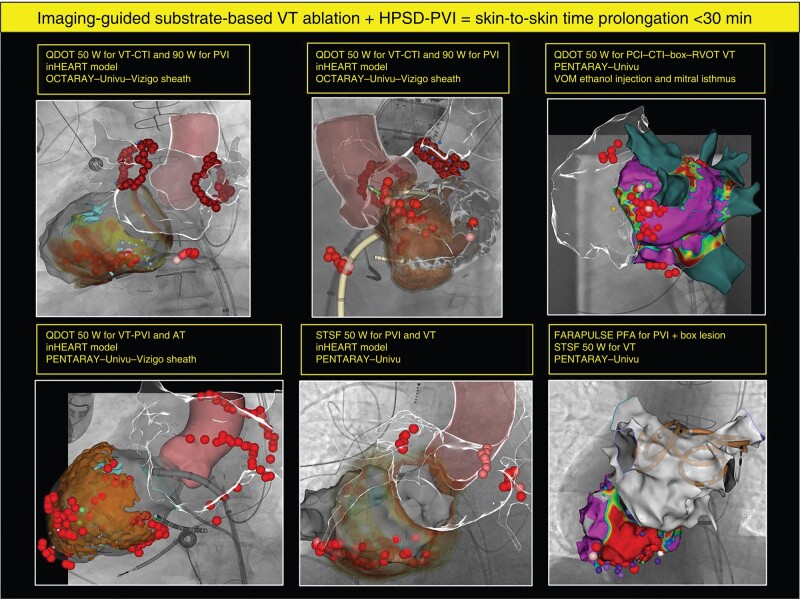
Examples of combined complex EP procedures. Six examples of combined atrial and ventricular ablation procedures using a 3D mapping system (CARTO, Biosense Webster), a substrate model of the patients’ ventricular scar (inHEART), a dedicated mapping catheter (PENTARAY or OCTARAY, both Biosense Webster), and an ablation catheter (QDOT, Biosense Webster, and/or FARAPULSE, Boston Scientific).

After a mean follow-up of 8.1 ± 8.3 months, six patients were free of any atrial or ventricular arrhythmia. One patient underwent cardioversion for atypical atrial flutter. Two patients had recurrence of sustained VT, one of which was terminated by ATP and the other one by administration of amiodarone. One patient had recurrent premature ventricular complexes. Recurrence rates did not differ significantly from the matched patients in the POWER-PLUS and MUSIC VT studies, respectively. No procedure-related complications were identified during follow-up.

The main findings of our study are as follows: (i) In experienced centres and well-selected patients, a combined VT ablation and PVI is feasible with an average combined procedure time of <3 h; (ii) with streamlined and efficient workflows, the increase in procedure time associated with atrial ablation procedures, including PVI, is <30 min; and (iii) despite the reduced cardiac reserve in patients undergoing VT ablation, combined VT and atrial ablation procedures do not appear to increase complication rates or compromise efficacy.

We demonstrate that a single ablation catheter can be used for ablation of not only multiple atrial arrhythmias but also VT during the same procedure. A very high-power short-duration AF ablation has a low risk for stroke and silent emboli and can be performed in a safe and efficient workflow as demonstrated in the FAST AND FURIOUS PVI Study.^6,11,12^ Additionally, a substrate-based VT ablation approach in sinus rhythm can be performed in around 2 h with a favourable safety profile.^2,13^ Due to increased standardization (CLOSE-PVI and substrate-based CT channel ablation in sinus rhythm), new ablation power strategies (50–90 W) and additional tools as preprocedural imaging, and multipolar mapping catheters for efficient high-density mapping and specific pacing manoeuvres, both AF and VT procedures became shorter and more predictable.^6,12,14,15^ However, our findings need to be validated in larger studies, with potential additional data on resource utilization and patient satisfaction. Finally, it is important to note that while the use of standardized AF ablation strategies is widespread, there remains heterogeneity in terms of VT ablation strategies. For instance, the use of inHEART image integration-based VT ablation is currently limited to specific centres, and the data supporting the approach are limited to single-centre studies.

## Data Availability

All relevant data appear in the manuscript. Other data are available upon reasonable request at Benjamin.berte@cardiopuls.ch.

## References

[1] Berte B , Kobza R, Toggweiler S, Schupfer G, Duytschaever M, Hoop V et al Improved procedural efficiency of atrial fibrillation ablation using a dedicated ablation protocol and lean management. JACC Clin Electrophysiol 2021;7:321–32. 33632635 10.1016/j.jacep.2020.08.023

[2] Berte B , Cochet H, Dang L, Mahida S, Moccetti F, Hilfiker G et al Image-guided ablation of scar-related ventricular tachycardia: towards a shorter and more predictable procedure. J Interv Card Electrophysiol 2020;59:535–44. 31858334 10.1007/s10840-019-00686-w

[3] Berte B , Purerfellner H. Efficiency as a new paradigm in electrophysiology: a lean approach within an agile mindset. Europace 2022;24:1716–7. 35640910 10.1093/europace/euac076

[4] Boehmer AA , Summ A, Vila S, Rothe M, Nussbaum E, Zezyk C et al Process optimization for atrial fibrillation ablation. Europace 2022;24:1763–8. 35989514 10.1093/europace/euac048

[5] Fernandez-Armenta J , Soto-Iglesias D, Silva E, Penela D, Jauregui B, Linhart M et al Safety and outcomes of ventricular tachycardia substrate ablation during sinus rhythm: a prospective multicenter registry. JACC Clin Electrophysiol 2020;6:1435–48. 33121673 10.1016/j.jacep.2020.07.028

[6] Heeger CH , Sano M, Popescu SS, Subin B, Feher M, Phan HL et al Very high-power short-duration ablation for pulmonary vein isolation utilizing a very-close protocol—the FAST AND FURIOUS PVI study. Europace 2023;25:880–8. 36546582 10.1093/europace/euac243 PMC10062369

[7] Hofer D , Steffel J, Hurlimann D, Haegeli L, Luscher TF, Duru F et al Long-term incidence of inappropriate shocks in patients with implantable cardioverter defibrillators in clinical practice-an underestimated complication? J Interv Card Electrophysiol 2017;50:219–26. 29177981 10.1007/s10840-017-0297-8

[8] Poole JE , Johnson GW, Hellkamp AS, Anderson J, Callans DJ, Raitt MH et al Prognostic importance of defibrillator shocks in patients with heart failure. N Engl J Med 2008;359:1009–17. 18768944 10.1056/NEJMoa071098 PMC2922510

[9] Powell BD , Saxon LA, Boehmer JP, Day JD, Gilliam FR 3rd, Heidenreich PA et al Survival after shock therapy in implantable cardioverter-defibrillator and cardiac resynchronization therapy-defibrillator recipients according to rhythm shocked. The ALTITUDE survival by rhythm study. J Am Coll Cardiol 2013;62:1674–9. 23810882 10.1016/j.jacc.2013.04.083

[10] O'Neill L , El Haddad M, Berte B, Kobza R, Hilfiker G, Scherr D et al Very high-power ablation for contiguous pulmonary vein isolation: results from the randomized POWER PLUS trial. JACC Clin Electrophysiol 2023;9:511–22. 36752467 10.1016/j.jacep.2022.10.039

[11] Calvert P , Kollias G, Purerfellner H, Narasimhan C, Osorio J, Lip GYH et al Silent cerebral lesions following catheter ablation for atrial fibrillation: a state-of-the-art review. Europace 2023;25:euad151. 37306314 10.1093/europace/euad151 PMC10259069

[12] Boga M , Suhai FI, Orban G, Sallo Z, Nagy KV, Szegedi L et al Incidence and predictors of stroke and silent cerebral embolism following very high-power short-duration atrial fibrillation ablation. Europace 2023;25:euad327. 37931067 10.1093/europace/euad327 PMC10653180

[13] Arenal A , Avila P, Jimenez-Candil J, Tercedor L, Calvo D, Arribas F et al Substrate ablation vs antiarrhythmic drug therapy for symptomatic ventricular tachycardia. J Am Coll Cardiol 2022;79:1441–53. 35422240 10.1016/j.jacc.2022.01.050

[14] Bhaskaran A , Fitzgerald J, Jackson N, Gizurarson S, Nanthakumar K, Porta-Sanchez A. Decrement evoked potential mapping to guide ventricular tachycardia ablation: elucidating the functional substrate. Arrhythm Electrophysiol Rev 2020;9:211–8. 33437489 10.15420/aer.2020.25 PMC7788395

[15] Mahida S , Sacher F, Dubois R, Sermesant M, Bogun F, Haissaguerre M et al Cardiac imaging in patients with ventricular tachycardia. Circulation 2017;136:2491–507. 29255125 10.1161/CIRCULATIONAHA.117.029349

